# VIBES: A multiscale modeling approach integrating within-host and between-hosts dynamics in epidemics

**DOI:** 10.1073/pnas.2523055123

**Published:** 2026-03-26

**Authors:** Paulo Cesar Ventura, Yong Dam Jeong, Maria Litvinova, Allisandra G. Kummer, Shingo Iwami, Hongjie Yu, Stefano Merler, Alessandro Vespignani, Keisuke Ejima, Marco Ajelli

**Affiliations:** ^a^Laboratory for Computational Epidemiology and Public Health, Department of Epidemiology and Biostatistics, Indiana University School of Public Health, Bloomington, IN 47405; ^b^interdisciplinary Biology Laboratory, Division of Biological Science, Graduate School of Science, Nagoya University, Nagoya 464-8602, Japan; ^c^Department of Mathematics, Pusan National University, Busan 46241, Republic of Korea; ^d^Department of Applied Mathematics, Pukyong National University, Busan 48513, Republic of Korea; ^e^Department of Epidemiology and Biostatistics, Indiana University, School of Public Health Bloomington, IN 47405; ^f^Shanghai Institute of Infectious Disease and Biosecurity, Fudan University, Shanghai 200032, China; ^g^School of Public Health, Fudan University, Key Laboratory of Public Health Safety, Ministry of Education, Shanghai 200032, China; ^h^Department of Infectious Diseases, Huashan Hospital, Fudan University, Shanghai 200040, China; ^i^Center for Health Emergencies, Bruno Kessler Foundation, Trento 38123, Italy; ^j^Laboratory for the Modeling of Biological and Socio-Technical Systems, Northeastern University, Boston, MA 02115; ^k^Lee Kong Chian School of Medicine, Nanyang Technological University, Singapore 308232, Singapore

**Keywords:** multiscale model, SARS-CoV-2, epidemiology, agent-based model

## Abstract

Infectious disease spread is a multiscale process composed of within-host (biological) and between-host (social) drivers and disentangling them from each other is a central challenge in epidemiology. Here, we introduce VIBES, a multiscale modeling framework that explicitly integrates viral dynamics based on patient-level data with population-level transmission on a data-driven network of social contacts. Using SARS-CoV-2 as a case study, we analyze three emergent epidemic properties, namely the generation time, serial interval, and presymptomatic transmission. First, we established a purely biological baseline, thus independent of the reproduction number (*R*), from the within-host model, estimating a generation time of 6.3 d for symptomatic individuals and 43.1% presymptomatic transmission. Then, using the full model incorporating social contacts, we found a shorter generation time (5.4 d at *R* = 3.0) and an increase in presymptomatic transmission (52.8% at *R* = 3.0), disentangling the impact of social drivers from a purely biological baseline. We further show that as pathogen transmissibility increases (*R* from 1.3 to 6), competition among infectious individuals shortens the generation time and serial interval by up to 21% and 13%, respectively. Conversely, a social intervention, like isolation, increases the proportion of presymptomatic transmission by about 30%. Our framework also estimates metrics that are challenging to obtain empirically, such as the generation time for asymptomatic individuals (5.6 d; 95%CI: 5.1 to 6.0 at *R* = 1.3). Our findings establish multiscale modeling as a powerful tool for mechanistically quantifying how pathogen biology and human social behavior shape epidemic dynamics as well as for assessing public health interventions.

## Significance

Predicting an epidemic’s course requires understanding how pathogen biology and human behavior independently shape transmission. While these scales are inextricably connected, traditional epidemiological methods often struggle to isolate biological traits from the influence of social contact patterns and public health interventions, limiting our ability to characterize novel pathogens. We introduce VIBES, a computational framework that integrates patient-level viral dynamics with data-driven contact patterns to bridge this gap. Our analysis quantifies how social contact patterns alter a pathogen’s biological baseline, accelerating the epidemic pace and expanding the window of silent presymptomatic transmission. By disentangling these drivers, VIBES provides a mechanistic foundation for identifying which transmission trends are biological versus behavioral, enabling better design of targeted public health interventions.

Understanding and predicting the course of an epidemic is a fundamental scientific challenge, primarily because infectious disease spread is a multiscale process. At one level is the within-host (biological) scale, where the replication dynamics of a pathogen and the host’s immune response jointly determine an individual’s outcomes (e.g., severity, infectiousness profile over the course of their infection) (1, 2). At another level is the between-hosts (social) scale, where human behavior, contact patterns, and social structures create the opportunities for transmission to occur (3, 4). These scales are inextricably interconnected (5), and a central objective of modern epidemiology is to disentangle their respective contributions in order to improve our ability to understand and predict epidemic trajectories and to design effective control measures.

Recent studies have leveraged viral load data collected from SARS-CoV-2 patients throughout the course of infection to develop mathematical models focused on viral replication and elimination within a single host. These models have been instrumental in characterizing SARS-CoV-2 viral load in individual cases of infection, providing critical insights into infectiousness profiles (6), guidelines for the implementation of screenings and isolation strategies (7–9), and the design of trials for antiviral drugs (10). However, they lack information about the contact network of infected individuals, which is key for the transmission dynamics of respiratory pathogens within a population (3, 11). This limits the capacity of within-host models to evaluate the effectiveness of a wide range of intervention options considered by public health authorities. Conversely, between-hosts mathematical models focus on pathogen transmission between infected individuals and their susceptible contacts (12). Such models have been widely used to improve our epidemiological understanding of spreading patterns and to evaluate the effectiveness of interventions (13, 14). However, they often lack the resolution to account for individual-level heterogeneities, such as differences in the clinical course of infection and viral shedding patterns, which shape epidemiological trends and the effectiveness of interventions (15). Such simplification makes it difficult to mechanistically connect what happens inside the host to the transmission patterns observed at the population level, thereby limiting our ability to disentangle the impact of biological and social drivers of the epidemic dynamics.

A recent literature review (5) on linking within-host and between-host scales identified several studies that have contributed to the theoretical foundations of a multiscale framework. However, few incorporated empirical virological data, and none integrated social contact data. To address this gap, we developed VIBES (Viral dynamics Individual-Based Epidemic Simulator), a multiscale framework that explicitly integrates within-host viral dynamics based on viral dynamics data with between-host transmission dynamics using a social network derived from data on in-person human contact patterns. The within-host component simulates viral kinetics using differential equations whereas the between-hosts component uses an agent-based model that stochastically simulates transmission among a synthetic population structured to incorporate main transmission settings for respiratory pathogens such as households, schools, and workplaces. This allows critical epidemiological characteristics to emerge naturally from the simulation’s core biological mechanisms and social interactions rather than being assumed upfront.

To illustrate the model’s capability to disentangle these components, we apply VIBES to the transmission dynamics of the ancestral SARS-CoV-2 lineage. We focus on three epidemiological metrics of high public health relevance, all sensitive to the interaction between viral kinetics and social contact i) the generation time, the interval between infection events in an infector–infectee pair; ii) the serial interval, the interval between symptom onsets in successive case generations; and iii) the proportion of presymptomatic transmission, the fraction of secondary infections occurring during the presymptomatic phase of the primary case. By simulating epidemic spread under various conditions, our study quantifies how pathogen-specific traits and population-level social dynamics independently shape the emergent properties of epidemic dynamics.

## Results

### The Multiscale Framework.

VIBES is a multiscale, agent-based model of SARS-CoV-2 transmission that combines: the viral replication of the pathogen within a single individual (within-host model) and the transmission of the pathogen between individuals (between-hosts model).

• The within-host model is based on a system of differential equations simulating viral replication and elimination within each infected agent where the joint posterior distributions of model parameters were estimated based on data from 210 SARS-CoV-2 infected individuals (16). The within-host model simulates individual trajectories of the viral load over the course of the infection.
• The between-hosts model leverages a synthetic population of agents that is statistically equivalent to the population of IN, which is structured to reflect social contact patterns data within households, schools, workplaces, and the broader community (17). Transmission of the pathogen between infected and susceptible synthetic individuals occurs in four social settings: households, schools, workplaces, and the community.

When an agent is infected in the between-hosts model, an independent run of the within-hosts model is performed to calculate the viral load trajectory for that agent. This trajectory determines the infectiousness profile over time and the epidemiological status of the agent in the between-hosts model (i.e., latent, infectious, recovered, etc.), effectively integrating the biological and social drivers. Details of the models are reported in the *Methods* and the *SI Appendix*.

**Fig. 1.**
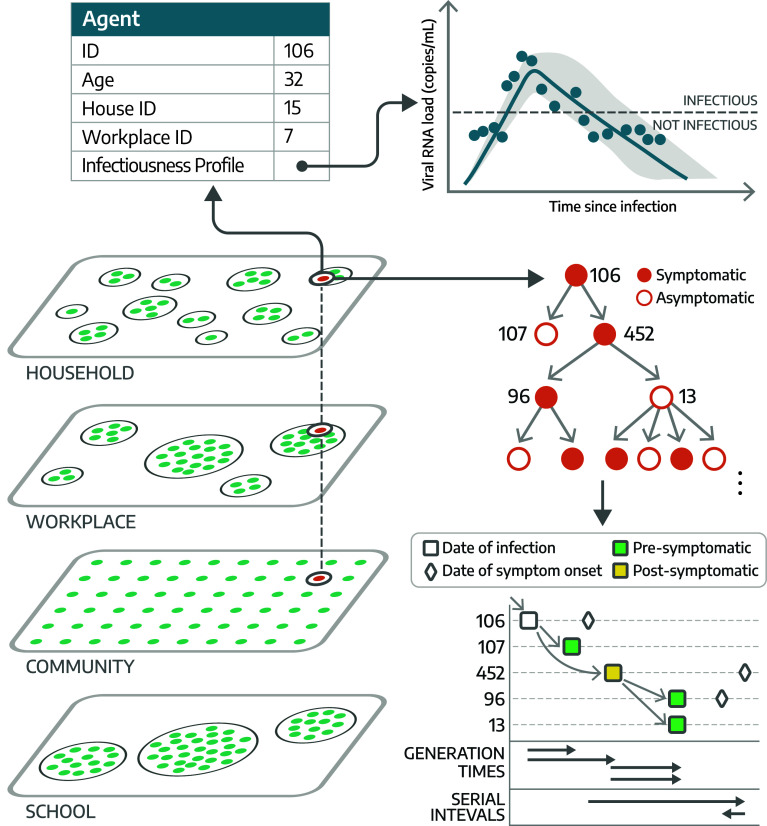
Conceptual representation of VIBES. We use a synthetic population of 500,000 agents, statistically equivalent to the population of Indiana (17). Transmission between individuals occurs in four settings: Households, schools, workplaces, and the community. The within-host model simulates the viral replication and elimination within each host, providing an individual viral load trajectory that determines the infectiousness profile and epidemiological status of each infected individual. By tracking individual infections, we directly calculate the following transmission metrics: i) generation time, defined as the time between the infection event of an infector and of their infectee(s); ii) serial interval, the time between the symptom onset of an infector and of their infectee(s); and iii) presymptomatic transmission, defined for symptomatic infectors as the proportion of secondary infections that occur before the symptom onset of the infector.

VIBES records the entire transmission chain (who infected whom) between the simulated agents and allows for the simulation of individual-level interventions. In the model, the pathogen’s transmissibility is calibrated to achieve a target reproduction number, (i.e., the number of secondary infections caused by a primary infector, R). Because the agent-based framework does not yield a closed-form expression for R, this calibration is performed by adjusting transmissibility parameters and numerically estimating R from simulated epidemic realizations, as described in the *Methods*. In this study, we modeled isolation of symptomatic individuals in their place of residence, as an example of an intervention that has traditionally been widely adopted at the onset of epidemic outbreaks. A schematic representation of VIBES is shown in Fig. 1; details on the model are reported in the *Methods* and *SI Appendix*.

To validate VIBES, we consider three key epidemiological metrics:

• The generation time, which is defined as the time interval between the infection event of an infector and their infectee(s). The generation time is a key determinant of the overall speed of epidemic growth with a shorter value resulting in a faster epidemic growth. In turn, this demands a rapid deployment of public health response to contain and/or mitigate an outbreak (18).
• The serial interval, which represents the time elapsed between the onset of symptoms in an infector and their infectee(s). The serial interval is important to inform contact tracing operation as well as isolation and quarantine guidelines. These types of nonpharmacological interventions are cornerstones of the public health response in the early phase of an outbreak when pharmacological options such as vaccines and antiviral treatments are not available (19).
• The presymptomatic transmission, which quantifies, for a symptomatic infector, the proportion of secondary infections that occur before the infector develops symptoms, relative to the total infections caused during their entire infectious period. Presymptomatic transmission poses a significant challenge for outbreak control, as it allows the pathogen to spread silently within a population. This highlights the need for measures that target transmission from individuals who are infectious but not yet showing symptoms such as widespread testing, mask-wearing, and social distancing (20).

These metrics were selected for this study because they are largely affected by pathogen transmissibility, individual infectiousness (which in VIBES is determined by the viral dynamics model), and human contact patterns (which may be affected by public health interventions). For example, as transmissibility increases, the generation time and serial interval become shorter due to an increase in competition between infectious individuals to infect susceptible individuals (21). This is particularly evident in social contexts with a limited number of individuals (e.g., households). Presymptomatic transmission increases as transmissibility increases due to the same mechanism. Regarding public health interventions, for example, when home isolation of identified infected individuals is in place, isolation shortens both the generation time and serial interval while it increases presymptomatic transmission.

It is important to stress that VIBES does not explicitly include the concept of the generation time, serial interval, and presymptomatic transmission (i.e., they are not model parameters). The combination of the within-host and between-hosts scales allows these metrics to emerge from the epidemic dynamics and be measured from the analysis of the transmission chain of simulated epidemics.

### Model Validation.

**Fig. 2.**
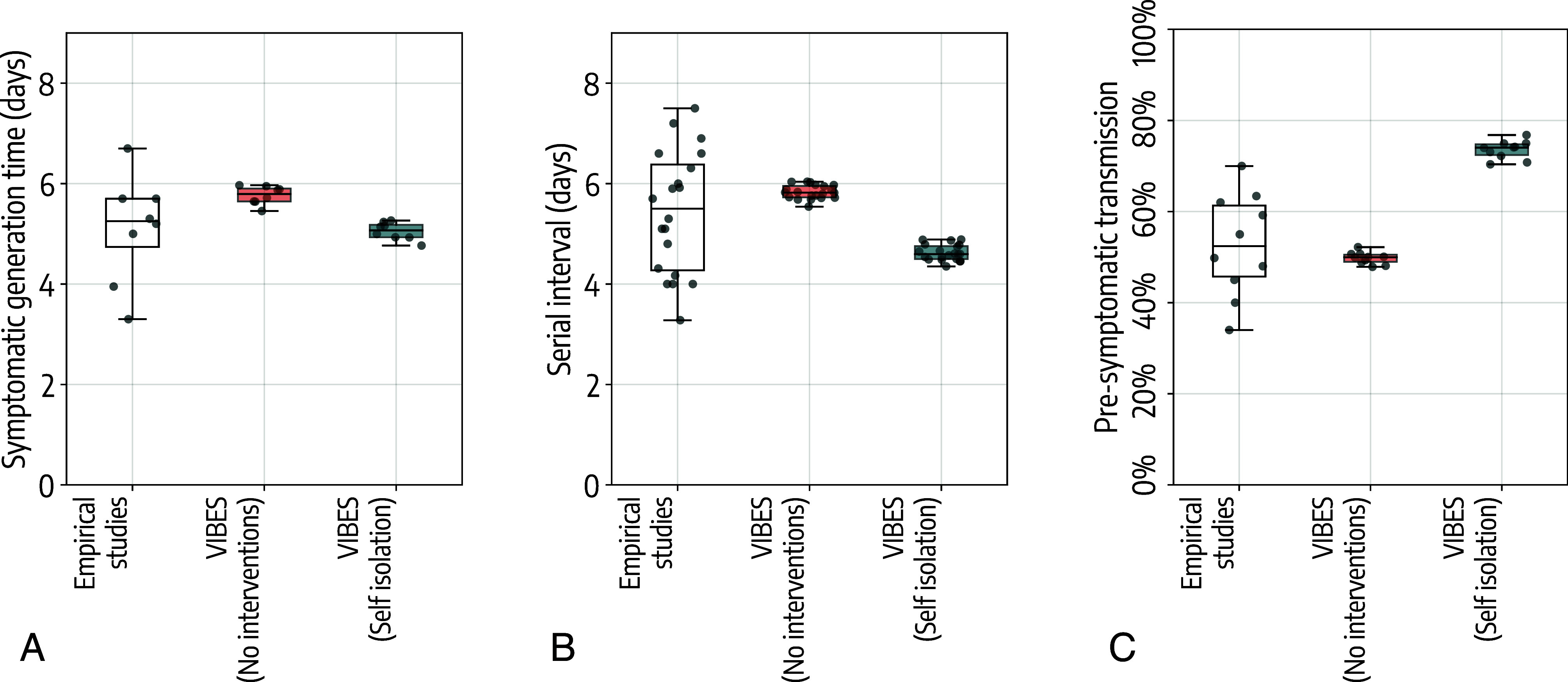
Estimated epidemiological metrics. (*A*) Boxplot of the distribution of the generation time for symptomatic individuals as estimated in independent empirical epidemiological studies (22–28) and as estimated by VIBES in the absence or presence of isolation of symptomatic individuals. In the identified epidemiological studies, the reproduction number was estimated in the range 1.3 to 2.8. For VIBES, we considered only scenarios based on the same values of the reproduction number reported in the identified epidemiological studies. The box represents the 50% interquantile range, the horizontal line represents the median and the whiskers indicate the farthest data point within 1.5 times the interquartile range from the box. Dots represent point estimates from each independent epidemiological study/simulated scenario. (*B*) As *A*, but for the serial interval. Empirical estimates were taken from refs. 23, 26, and 29–42, where the reproduction was estimated in the range 1.3 to 3.3. (*C*) As *A*, but for the proportion of presymptomatic transmission. Empirical estimates were taken from refs. 22, 23, 25–28, 34, and 36, where the reproduction was estimated in the range 1.3 to 2.8.

We validated model estimates against the ones obtained from the empirical studies (*Methods*). For the generation time, empirical studies estimated a mean generation time for the ancestral SARS-CoV-2 lineages of 5.1 d (95%CI: 3.4 to 6.5) for reproduction numbers between 1.3 and 2.8 (22–28). Leveraging the transmission chains simulated by VIBES and using the same range for the reproduction number found in the analyzed empirical studies, we estimated a mean symptomatic generation time (i.e., the infector is symptomatic) of 5.7 d (95%CI: 5.5 to 6.0) when no isolation policies are implemented and 5.0 d (95%CI: 4.8 to 5.2) when all symptomatic individuals are isolated at home after symptom onset (Fig. 2*A*).

For the serial interval, empirical studies estimated a mean value of 5.4 d (95%CI: 3.6 to 7.4) for reproduction numbers ranging from 1.3 to 3.3 (26, 29–32, 34, 36, 37, 39–42). From VIBES, we estimated a mean serial interval of 5.8 d (95%CI: 5.5 to 6.0) when no isolation policies are implemented and 4.6 d (95%CI: 4.3 to 4.9) when all symptomatic individuals are isolated at home after symptom onset (Fig. 2*B*).

For presymptomatic transmission, empirical studies estimated a mean of 52.6% (95%CI: 35.4 to 68.5) for reproduction numbers ranging from 1.3 to 2.8 (22, 23, 25–28, 34, 36). From VIBES, we estimated a mean of 50.1% (95%CI: 48.0 to 52.1) when no isolation policies are implemented and 74.9% (95%CI: 70.6 to 76.7) when all symptomatic individuals are isolated at home after symptom onset (Fig. 2*C*).

The larger variability observed in the empirical estimates as compared to our model-based estimates is associated with the heterogeneities between studies in terms of sample size (e.g., number of analyzed transmission events), pathogen transmissibility (reproduction number), implemented public health interventions, study location (social contacts and demographics), etc., with single studies often reporting multiple estimates for different time periods and locations (43, 44). Our model allows us to adjust for these heterogeneities by simulating transmission in a controlled setting (location, reproduction number, and intervention) to assess the effect of transmissibility and isolation on the generation time, serial interval, and presymptomatic transmission. Moreover, our model estimates were obtained by considering the entire transmission chain, which include from tens to hundreds of thousands of events depending on the analyzed scenario, as compared to a few tens up to a few hundred transmission events analyzed in the empirical epidemiological studies used for model validation (22–42). An analysis of the effect of sample size and deployed public health interventions is reported in the *SI Appendix*.

### Disentangling the Biological and Social Components of Transmission.

VIBES offers a level of control that allows the independent assessment of how social behavior and biological processes shape key epidemiological metrics. While the within-host model can estimate these metrics based solely on the biological interaction between pathogen and host, VIBES integrates this information with the effects of pathogen transmissibility, contact patterns, and interventions such as isolation. Details on how we estimated epidemiological metrics from the within-host model and with VIBES for a range of R values can be found in the *Methods*.

**Fig. 3.**
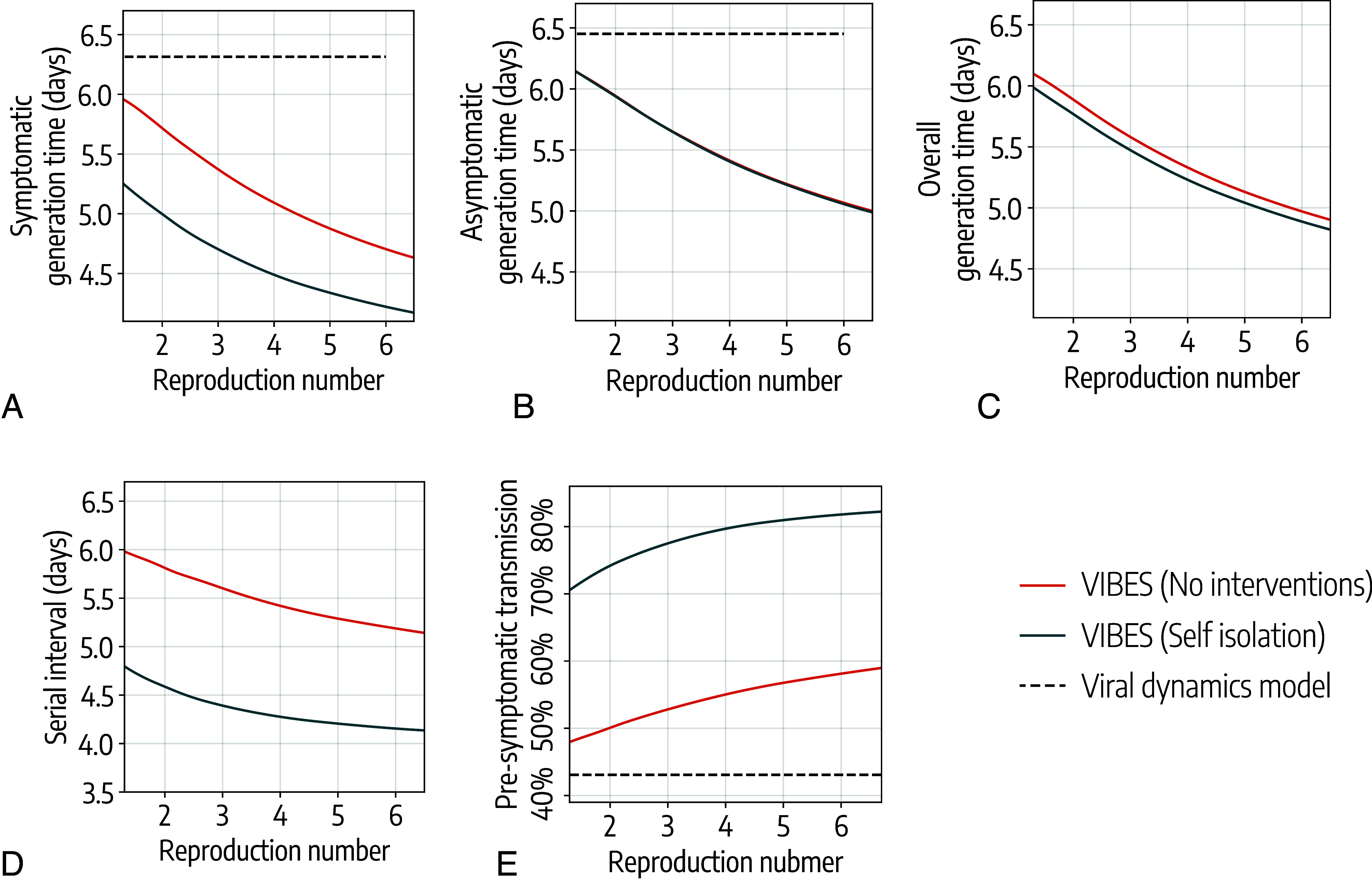
Effect of transmissibility and isolation on epidemiological metrics. (*A*) Average generation time for symptomatic individuals estimated by VIBES in the presence and absence of isolation of symptomatic individuals as a function of the reproduction number. The dashed line represents the estimated average generation time using the viral dynamics model only, which does not depend on the value of the reproduction number. (*B*) As *A* but for the generation time for asymptomatic individuals. (*C*) As *A* but for the generation time for all infected individuals. (*D*) As *A* but for the average serial interval. (*E*) As *A* but for the proportion of presymptomatic transmission.

To disentangle the biological from social effects, first, we ran the within-host model alone, which considers only biological factors. We obtained estimates for the mean generation time of 6.3 d for symptomatic and 6.5 d for asymptomatic individuals, irrespective of the reproduction number (Fig. 3 *A* and *B*). These estimates are derived using normalized infectiousness profiles obtained from the sampled viral load trajectories, as detailed in the *Methods*. Then, we ran VIBES, which considers both biological and social factors, obtaining lower estimates of the generation time. For a reproduction number of 3.0, VIBES estimates a generation time of 5.4 d (95%CI: 5.3 to 5.4) for symptomatic and 5.7 d (95%CI: 5.6 to 5.7) for asymptomatic individuals, a 14.3% and 12.3% decrease from the within-host model estimates, respectively (Fig. 3 *A* and *B*).

Estimates from VIBES also show a decreasing trend of the generation time with increasing transmissibility. Assuming no isolation policies, VIBES shows a 21.7% decrease in the mean generation time of symptomatic individuals with a reproduction number ranging from 1.3 to 6.0, going from 6.0 d (95%CI: 5.9 to 6.0) to 4.7 d (95%CI: 4.7 to 4.7) for reproduction numbers of 1.3 and 6, respectively (Fig. 3*A*). For asymptomatic individuals, VIBES shows a 16.4% decrease in generation time in the same range for the reproduction number, going from 6.1 d (95%CI: 6.1 to 6.2) to 5.1 d (95%CI: 5.1 to 5.1) (Fig. 3*B*). These lead to an estimate of the overall generation time that lies in between the two (Fig. 3*C*).

When considering home isolation for symptomatic individuals, we estimated a 20.7% decrease in the generation time for symptomatic individuals as the reproduction number increases, going from 5.3 d (95%CI: 5.2 to 5.3) to 4.2 d (95%CI: 4.2 to 4.2) from a reproduction number of 1.3 to 6.0 (Fig. 3*A*). Since we assumed no isolation of asymptomatic individuals, the estimated generation time for asymptomatic infectors is not affected by the intervention (Fig. 3*B*) and only a marginal effect is found for the overall estimate (Fig. 3*C*).

The serial interval showed a similar decreasing trend for increasing transmissibility. Using VIBES and assuming the absence of isolation policies, we estimated that the serial interval decreases from 6.0 d (IQR: 5.9 to 6.1) to 5.2 d (95%CI: 5.2 to 5.2) as the reproduction number increases from 1.3 to 6, respectively, corresponding to a 13.3% decrease (Fig. 3*D*). When considering 100% isolation of symptomatic individuals, we estimated a decrease in the serial interval from 4.8 d (IQR: 4.7 to 4.9) to 4.2 d (IQR: 4.1 to 4.2) as the reproduction number increased from 1.3 to 6.0, respectively, corresponding to a 12.5% decrease. When isolation is considered, the estimated serial interval is about 20% shorter than that estimated in the absence of isolation, regardless of the value of the reproduction number (Fig. 3*D*).

Again, to disentangle the biological and social factors, first, we run the within-host viral dynamics model alone, finding a 43.1% fraction of presymptomatic transmission. Using VIBES, assuming a reproduction number R=3.0 and in the absence of isolation, we estimated a presymptomatic transmission of 52.8% (95%CI: 52.4 to 53.3), which is 22.5% higher than the estimate that considers only biological drivers.

As transmissibility increases, more presymptomatic transmission is observed. Using VIBES and having no isolation policies in place, we estimated a 17.3% increase in presymptomatic transmission, ranging from 48.0% when R=1.3 to 58.1% when R=6 (Fig. 3*E*). Isolation of symptomatic individuals has a marked effect on presymptomatic transmission that increases to 70.5% when R=1.3 and to 81.8% when R=6 (Fig. 3*E*).

We conducted a set of sensitivity analyses to assess the robustness of our results to different modeling assumptions. We found that our results are only marginally affected by changes in the probability of developing symptoms and the infectiousness of symptomatic individuals relative to asymptomatic ones (*SI Appendix*, Figs. S6 and S7). Small quantitative differences were observed when infectiousness was homogeneous between individuals (i.e., no superspreading), although the qualitative trends were confirmed (*SI Appendix*, Fig. S8).

## Discussion

We validated the VIBES model against empirical epidemiological data for the ancestral lineage of SARS-CoV-2, showing good agreement between our model-based estimates of key epidemiological metrics and those obtained from traditional field epidemiological studies. By combining individual-level viral load data with detailed synthetic contact networks, VIBES provides insights into epidemiological characteristics that are challenging to measure in the field. Specifically, we estimated the generation time for asymptomatic individuals to be 5.6 d (95%CI: 5.1 to 6.0 d) when R=1.3. Our study also characterizes the associations between pathogen transmissibility, interventions, and the key epidemiological metrics. Specifically, we found that with increasing pathogen transmissibility (from R=1.3 to 6), the generation time and serial interval decreased by 21% and 13%, respectively, which is compatible with findings from field studies showing a progressive shortening of these two indicators as new more transmissible variants emerged (45). Moreover, the contribution of presymptomatic transmission increased by 17% (R from 1.3 to 6). Isolation of symptomatic individuals shortened both generation time and serial interval by about 2% and 20%, respectively, and increased the proportion of presymptomatic transmission by about 30%. These findings show the complex interplay between pathogen transmissibility, individual-level interventions, and key epidemiological metrics that shape outbreak dynamics.

A strength of VIBES is its ability to estimate critical epidemiological metrics from the early stages of an outbreak, drawing on data sources that are potentially available in real time. From the one side, VIBES relies on individual-level viral load data, which can be approximated from PCR testing that often becomes available early in an outbreak, as shown by the deployment of PCR diagnostics within two weeks since the admission of the first COVID-19 patients in a Wuhan hospital (29, 46, 47). From the other side, VIBES relies on contact pattern data that can be derived from preexisting sources, including publicly available micro- and macrolevel datasets, social contact studies, and synthetic populations representing specific locations (17, 48–53). Alternatively, contact patterns can be approximated using mobility data collected in real time (54–57). It is important to emphasize that our approach is not intended to replace traditional epidemiological studies but to complement them by providing an additional analytical tool for understanding the spread of novel pathogens. Indeed, estimating metrics like the generation time (especially for asymptomatic individuals) and the fraction of presymptomatic transmission poses several challenges in traditional field studies. Empirical estimates also exhibit larger variability due to heterogeneities in study populations, social behaviors, limited sample size, and implemented interventions resulting in wide CI and making results of different studies often hard to reconcile (45, 58–61). VIBES, however, can derive these estimates in a controlled setting from first principles (i.e., they are emerging properties of the model).

The epidemiological metrics used in this study (i.e., generation time, serial interval, and proportion of presymptomatic transmission) are crucial for understanding the epidemiology of novel pathogens and designing effective control measures. For instance, the generation time dictates the pace of the epidemic, providing information on the timeline of deployment of public health interventions (18). The serial interval provides insights into the design of symptom-based interventions such as case isolation (19). The fraction of presymptomatic transmission provides information about the need for interventions that go beyond symptom-based approaches. However, it is important to stress that other key transmission parameters and epidemiological metrics (e.g., reproduction number, infectiousness of symptomatic relative to asymptomatic infectors, infection fatality risk, infection hospitalization risk) are also crucial for preparedness and response planning (62, 63). While the relative infectiousness was incorporated into our modeling framework and explored through sensitivity analyses, it was not among the metrics estimated in this study. Overall, this emphasizes the importance of using a range of complementary approaches during an epidemic outbreak to provide timely and reliable insights into its epidemiology, transmission dynamics, and public health impact.

It is important to acknowledge the limitations of our study. First, our analysis uses the ancestral lineage of SARS-CoV-2 as a case study. While the identified qualitative trends may be common to other pathogens, the quantitative estimates obtained in this study could hardly be generalized to other SARS-CoV-2 variants or other pathogens. Second, the within-host model of viral dynamics is directly taken from our previous study (16). While this can be seen as a strength of this study, as it relies on a well-established and validated approach, other viral dynamics models have been introduced in the literature considering more complex viral dynamics [e.g., considering an eclipse phase slowing down viral growth (64) or an innate immune response—interferons (37)]. A new version of VIBES could benefit from more refined within-host models. Third, VIBES, in its current form, relies on a simplified representation of human behavior and does not account for potential changes in social contact patterns or adherence to interventions over time. These can affect both qualitative trends and quantitative estimates of epidemiological metrics. Moreover, our analysis considers only one type of intervention: isolation of symptomatic individuals. While isolation is a crucial control measure, it is just one component in a wide array of public health interventions considered by policy makers. For instance, antiviral treatment can reduce viral load of treated patients, which may in turn lower their transmission potential. Finally, our analysis focuses only on a single geographic location, using a synthetic population representative of IN. While we expect the identified patterns to hold true for other locations, quantitative estimates may be different.

In conclusion, we introduced a multiscale modeling framework, VIBES, that bridges the gap between within-host viral dynamics and population-level transmission, disentangling the influence of biological and social drivers of transmission. By providing a mechanistic understanding of key epidemiological metrics and their interplay with transmissibility and interventions, VIBES offers a valuable tool for informing public health strategies.

## Methods

### Within-Host Model.

For this study, we developed the viral dynamics model presented in Jeong et al. (16). Briefly, the model consists of a set of two ordinary differential equations accounting for the fraction of uninfected target cells and the number of viruses per unit of sample specimens (i.e., viral load in copies/ml). A nonlinear mixed effects model was used to fit empirical viral load data retrieved from 210 patients infected with the ancestral SARS-CoV-2 lineage (109 symptomatic, and 101 asymptomatic) to estimate the posterior distribution of three free model parameters. The procedure was independently performed for symptomatic and asymptomatic patient data, resulting in different parameter distributions. The infectiousness profile was assumed to be proportional to the logarithm of the viral load (copies/ml) using a threshold of 10^5.0^ copies/ml, under which an individual is not able to transmit the virus (65, 66). For symptomatic infections, the incubation period (time from infection to symptom onset) was estimated from the viral load to match the empirical distribution estimated in Hu et al. (23), for which an average of 6.4 d (IQR: 3.2 to 8.8) was found. Details on the viral dynamics model and model fitting procedure can be found in the *SI Appendix*.

### VIBES.

In our model, transmission between infectious and susceptible individuals takes place among a synthetic population of agents that can be represented as a multiplex layer network with four layers: household, school, workplace, and general community. The synthetic population has been taken from our previous work (17), and it comprises 200,000 households (approximately 500,000 individuals) representative of the IN population. Briefly, each individual in the synthetic population is assigned to a household and the general community. Based on employment and school enrollment rates by age, each individual is assigned to a workplace and/or a school (if any) in the respective layers. Homogeneous mixing is assumed within each household, workplace, school, and in the general community.

The transmission model features six epidemiological statuses to which each individual is assigned at each step of the simulation: susceptible, latent, infectious presymptomatic, infectious symptomatic, infectious asymptomatic, and removed. Susceptible individuals can contract the infection through contact with an infected individual. Upon infection, we use an age-dependent probability (67) to sample whether the individual will develop symptoms or remain asymptomatic. Then, a viral load profile is generated by the viral dynamics model, where the parameters are sampled from the distribution fitted for symptomatic or asymptomatic patients separately. At the first simulation step after the simulated viral load exceeds the infectiousness threshold, the (latent) individual becomes able to transmit the virus; when the threshold is crossed once again in the declining phase of the infection, the individual is no longer able to transmit the virus and is considered to be removed.

The transmission probability $p_{i\to j}\left(t\right)$ that infectious individual i infects susceptible individual j that is in contact with i in setting $\begin{array}{c} l\in \{\text{household}, \end{array}$$\begin{array}{c} \text{workplace},\text{school},\text{community}\} \end{array}$ at time t is defined as

$$p_{i\to j}\left(t\right)=\alpha \cdot \frac{\beta_{l}}{N_{i,l}}\cdot g_{i}\cdot \psi_{i}\cdot u_{i}\left(t-\tau_{i}\right)\cdot \Delta t,$$

where

 *β_l_* is the setting-specific transmission risks, which were estimated to match the proportions of infections by social setting reported in Liu et al. (13);
• α is a scaling rate that is set to obtain the desired value of the reproduction number;
• *N_i,l_* is the number of individuals in setting *l* to which individuals *i* and *j* belong;
• *τ_i_* is the time when individual *i* was infected;
• $u_{i}\left(t-\tau_{i}\right)$ is the infectiousness profile of individual *i* at step $t-\tau_{i}$, determined by the viral dynamics model (see *SI Appendix* for details);
• $g_{i}$ is a coefficient determining the overall infectiousness of individual *i*, which is sampled from a gamma distribution with shape = 0.235 and scale = 4.26 as estimated in Sun et al. (26);
• $\psi_{i}$ is the infectiousness of symptomatic individuals relative to asymptomatic individuals; namely, $\psi_{i}=1$ if *i* is asymptomatic and $\psi_{i}=\psi$ if *i* is symptomatic. ψ=1 in the baseline analysis, while the values up to 5 are explored in a sensitivity analysis.
• Δt = 0.25 d is the time step of the simulation.

We also implemented a 100% isolation policy for symptomatic individuals, who isolate themselves in their place of residence since the day of symptom onset until the end of the infectious period. When an individual is isolated, they continue to have contacts with their household members, while they do not have any contacts with individuals in other social settings.

Details on the between-hosts transmission model are reported in the *SI Appendix*.

### Estimation of Epidemiological Metrics Using VIBES.

During the simulation, we track the entire transmission chains and produce a simulated line list of patients that contains information about the infector, infection date, and date of symptom onset. From this line list, we calculated epidemiologically relevant metrics. Specifically

• The reproduction number was calculated as
  $$R=\frac{1}{M_{W}}\sum_{i|t_{i}\in W}r_{i},$$
  where W is a subset of all time steps of a simulation where the infection incidence shows an exponential growth (Details in the *SI Appendix*), $r_{i}$ is the number of secondary infections caused by individual i, $t_{i}$ is the time in which individual i was infected, $M_{W}=\sum_{i|t_{i}\in W}1$ is the number of individuals that were infected in time window W, and $i|t_{i}\in W$ selects only individuals that were infected in time window W.
• The serial interval *S_I_* is calculated as
  $$S_{I}=\frac{1}{N_{F}}\sum_{e\in F}(\tau_{e}-\sigma_{e}),$$
  where F is the set of infector–infectee pairs in which both individuals develop symptoms, $\tau_{e}$ is the time at which the infected individual developed symptoms, $\sigma_{e}$ is the time at which the infector individual developed symptoms, and $N_{F}$ is the number of elements in F.
• The overall generation time *T_G_* was calculated as:
  $$T_{g}=\frac{1}{N_{E}}\sum_{e\in E}(t_{e}-s_{e}),$$
  where E is the set of all infector–infectee pairs, $t_{e}$ is the time at which the infection occurred, $s_{e}$ is the time at which the infector was infected, and $N_{E}$ is the number of infections during the simulation. Similarly, we defined the symptomatic generation time and asymptomatic generation time by limiting the set E to include only symptomatic or asymptomatic infectors, respectively.
• The fraction of presymptomatic transmission *P_T_* was calculated:
  $$P_{T}=\frac{1}{N_{G}}\sum_{e\in G}J(t_{e}<\sigma_{e}),$$
  where G is the set of infector–infectee pairs in which the infector is symptomatic, $N_{G}$ is the number of elements in set G, and J is an indicator variable that is 1 if the condition is true, 0 otherwise.

### Estimation of Epidemiological Metrics Using the Viral Dynamics Model.

We used the within-host component of VIBES to estimate epidemiologically relevant metrics directly from the simulated viral trajectories. Specifically

• The generation time was estimated as
  $$T_{g}=\frac{1}{N_{P}}\sum_{k\in P}\sum_{t=0}^{\infty }u_{k}\left(t\right)\cdot t,$$
  where P is the set of viral load profiles of either symptomatic or asymptomatic patients, $N_{P}$ = 10,000 is the number of samples and $u_{k}\left(t\right)$ is the normalized infectiousness from the sampled viral trajectory k at time t measured after infection, so that $\sum_{t=0}^{\infty }u_{k}\left(t\right)=1,\forall k\in P$.
• The proportion of presymptomatic transmission was estimated as
  $$P_{T}=\frac{1}{N_{P}}\sum_{k\in P}\sum_{t=0}^{\tau_{k}-1}u_{k}\left(t\right),$$
  where again P is the ensemble of viral load profile for symptomatic individuals and $\tau_{k}$ is the incubation period for sampled viral load profile k.

### Literature Estimates of Epidemiological Metrics.

For validation and comparison with the VIBES model, we collected estimates of the generation time, serial interval, and presymptomatic transmission from a systematic literature review (45). Among the papers cited in the literature review, we selected only those that i) provide at least one estimate of the generation time, serial interval, or fraction of presymptomatic transmission for the ancestral SARS-CoV-2 lineage, and ii) provide an estimate of the reproduction number that is greater than 1, ensuring that the metrics were estimated in the growing phase of an outbreak.

## Supplementary Material

Appendix 01 (PDF)

## Data Availability

Model simulations were performed on a standard laptop, with runtimes ranging from 20 to 90 s per scenario. To support the reproduction and extension of our work, the full C + + codebase for VIBES, together with a user and set up guide, is openly available on GitHub at https://github.com/CEPH-Lab/vibes-model-project. Previously published data were used for this work [Synthetic population from: Mistry et al. (17). Longitudinal viral load data from: Jeong et al. (16)].
