# Supplementary material for: VIBES: A multiscale modeling approach integrating within-host and between-hosts dynamics in epidemics
Source: Proc Natl Acad Sci U S A. 2026 Mar 26;123(13):e2523055123. doi: 10.1073/pnas.2523055123 (PMC13037879; doi:10.1073/pnas.2523055123)
Supplement: Supplementary file 1 — Appendix 01 (PDF) [file pnas.2523055123.sapp.pdf]

PNAS

Supporting information for

VIBES: A Multi-Scale Modeling Approach Integrating Within-Host and Between-Hosts Dynamics in Epidemics

Paulo C. Ventura[1], Yong Dam Jeong[2,3,4], Maria Litvinova[5], Allisandra G. Kummer[1], Shingo Iwami[2], Hongjie Yu[6,7,8], Stefano Merler[9], Alessandro Vespignani[10], Keisuke Ejima[11], Marco Ajelli[1,*]

[1]Laboratory for Computational Epidemiology and Public Health, Department of Epidemiology and Biostatistics, Indiana University School of Public Health, Bloomington, IN, USA

[2]interdisciplinary Biology Laboratory (iBLab), Division of Biological Science, Graduate School of Science, Nagoya University, Nagoya, Japan

[3]Department of Mathematics, Pusan National University, Busan, Republic of Korea

[4]Department of Applied Mathematics, Pukyong National University, Busan, Republic of Korea

[5]Department of Epidemiology and Biostatistics, Indiana University School of Public Health, Bloomington, IN, USA

[6]Shanghai Institute of Infectious Disease and Biosecurity, Fudan University, Shanghai, China

[7]School of Public Health, Fudan University, Key Laboratory of Public Health Safety, Ministry of Education, Shanghai, China

[8]Department of Infectious Diseases, Huashan Hospital, Fudan University, Shanghai, China

[9]Center for Health Emergencies, Bruno Kessler Foundation, Trento, Italy

[10]Laboratory for the Modeling of Biological and Socio-technical Systems, Northeastern University, Boston, Massachusetts, USA

[11]Lee Kong Chian School of Medicine, Nanyang Technological University, Singapore, Singapore

*Corresponding author: majelli@iu.edu

Table of Content

Methods **..........2**

Viral dynamics model 2

Fitting procedure 2

Conversion of viral load to infectiousness (and infectiousness threshold) 4

Simulation of incubation period using viral dynamics model 5

Synthetic population 6

VIBES model for SARS-CoV-2 transmission 6

Time window for the estimation of the reproduction number 8

Supplementary Results 9

Estimation of uncertainty 9

Epidemic trajectories 12

Sensitivity analysis: Probability of symptom development 13

Sensitivity analysis: Infectiousness of symptomatic individuals relative to asymptomatic ones 14

Sensitivity analysis: Distribution of the numbers of secondary infections 15

References 16

Methods

Viral dynamics model

Longitudinal viral load data was taken from Jeong et al.[1], which includes samples for 210 patients who met the criteria (109 symptomatic and 101 asymptomatic patients). We used a simple viral dynamics model to describe the evolution of the viral load over the course of infection. This model has been successfully employed for infectious diseases that cause infection, including SARS-CoV-2[2,3]:

$$\frac{df(t)}{dt} = -\beta f(t)V(t), \qquad (1)$$

$$\frac{dV(t)}{dt} = \gamma f(t)V(t) - \delta V(t). \qquad (2)$$

The first variable $f(t)$ is the ratio between the numbers of uninfected target cells at time t and 0. The second variable $V(t)$ is the number of viruses per unit of sample specimens (copies/ml) at time t. The parameters in the model, β, γ, and δ are the rate of virus infection, the maximum rate of viral replication, and the virus clearance rate of infected cells, respectively. The time t is measured in days, with $t = 0$ representing the day of infection. Following the previous study from Goyal et al. [4], the viral load at the day of infection was set as $V(0) = 10^{-2}$ (copies/ml), and $f(0) = 1$ by definition. Under reasonable parameter setting, the time course change of viral load $V(t)$ typically presents a bell-shaped curve (see Fig. S1); the viral load exponentially increases after infection, hits the peak, and starts declining once a substantial number of cells were infected, which is empirically observed in longitudinal viral load data that cause acute infections.

Fitting procedure

We employed a nonlinear mixed-effects model[5] for fitting the viral dynamics model to longitudinal viral load data using the software MONOLIX 2019R2[6].

The nonlinear mixed-effects model incorporates two separate components for each parameter: a fixed effect and a random effect. The fixed effects, $\theta = (\beta, \gamma, \delta)$, capture the typical population-level dynamics. To capture between-individual heterogeneity, we introduce individual-level random effects $\eta_i = (\eta_{\beta,i}, \eta_{\gamma,i}, \eta_{\delta,i})$ for each individual i. The corresponding individual-level parameter vector, $\theta_i = (\beta_i, \gamma_i, \delta_i)$, is given by

$$\beta_i = \beta e^{\eta_{\beta,i}}, \qquad \gamma_i = \gamma e^{\eta_{\gamma,i}}, \qquad \delta_i = \delta e^{\eta_{\delta,i}}.$$

Therefore, we have $\theta_i = \theta \odot e^{\eta_i}$, where $\odot$ denotes element-wise multiplication.

Under this parameterization, each individual-level parameter θ_i follows a log-normal distribution where the median is equal to the fixed effect, ensuring the positivity of the parameters. The random effects η_i are assumed to be independent across individuals and identically distributed draws from a multivariate normal distribution with zero mean and a population covariance matrix Ω, such that $\eta_i \sim N(0, \Omega)$. While η_i represents the unique deviation of individual i, the matrix Ω is a population hyperparameter that characterizes the magnitude of variability across the entire cohort.

Each individual parameter follows a log-normal distribution with population mean equal to the fixed effect and a multiplicative random deviation ensuring the positivity of the parameters. The random effects, η_i, are assumed to be normally distributed with zero mean and covariance matrix Ω, $\eta_i \sim N(0, \Omega)$.

The population parameters $\theta = (\beta, \gamma, \delta)$, and Ω were estimated using Stochastic Approximation Expectation Maximization (SAEM), while individual parameters $\theta_i = (\beta_i, \gamma_i, \delta_i)$, which represent the combination of fixed and random effects via the log-normal parameterization $\theta_i = \theta \odot e^{\eta_i}$, were obtained using Markov Chain Monte Carlo (MCMC). Because of the complex landscape of the likelihood functions, which requires high-dimensional parameter space exploration, the estimation procedures were carried out in two steps: the first step is the exploration phase, and the second step is the smoothing phase. In brief, the parameter region with maximum likelihood was explored and identified in the first step, then the identified parameter region was smoothed to identify the maximum likelihood estimate in the second step. Individual parameters were estimated as empirical Bayes estimates (EBEs). The fitting procedure was conducted for symptomatic and asymptomatic patients separately.

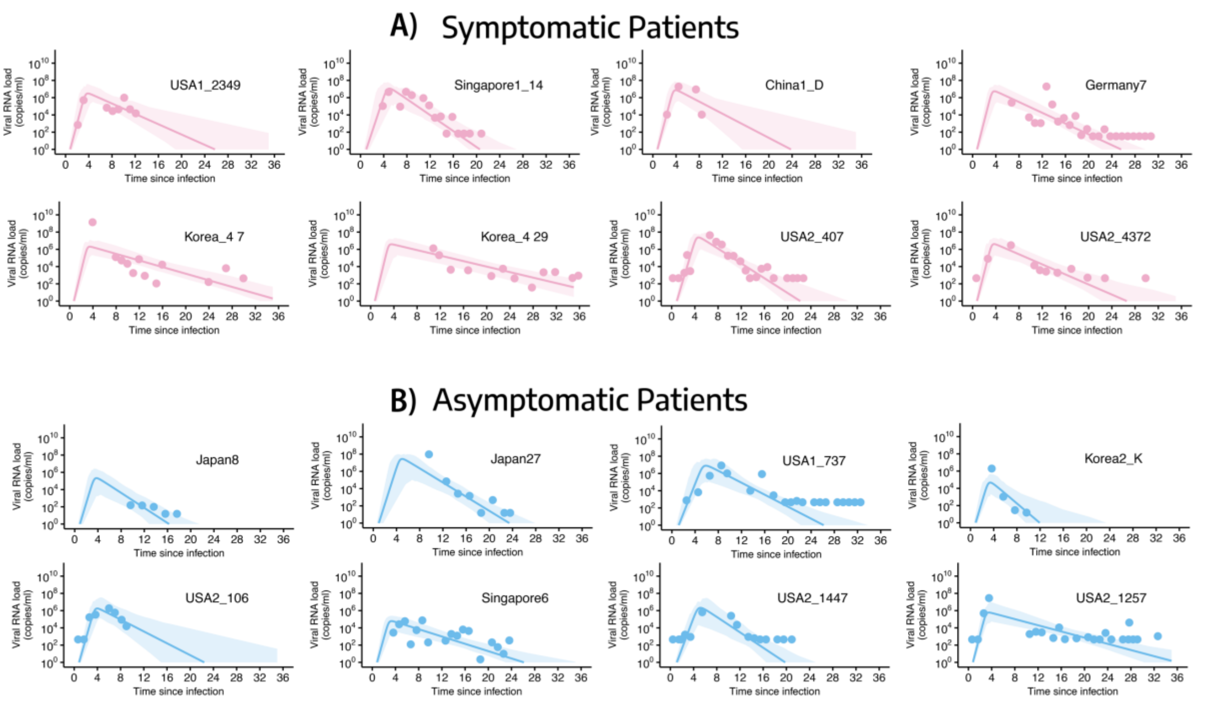

Figure S1. Samples from the longitudinal viral load data and the viral dynamics model fit. A Eight examples of longitudinal viral load (circles) randomly selected from the symptomatic patients in Jeong et al.1. The time on the x-axis is measured from the day when the patient was infected. The solid line represents the fitted viral dynamics trajectory obtained using the individual parameter estimates, and the shaded area represents the 95% prediction interval of the model-predicted viral load. B Same as A for eight randomly selected asymptomatic patients. Parameter values for these 16 patients are reported in Table S1.

Fig. S1 shows the estimated viral load dynamics for eight randomly selected symptomatic patients and eight asymptomatic ones. Table S1 reports the estimated individual-level parameters $\theta_i = (\beta_i, \gamma_i, \delta_i)$ for each of these patients. Fig. S2 summarizes the estimated distributions of model parameters when considering the entire population sample of 109 symptomatic and 101 asymptomatic patients.

Table S1. Individual parameter estimates for the 16 patients shown in Fig. S1.

	Patient ID (i)	β_i	γ_i	δ_i		Patient ID (i)	β_i	γ_i	δ_i
Symptomatic patients	USA1_2349	$1.39 \cdot 10^{-6}$	6.48	0.69	Asymptomatic patients	Japan8	$1.57 \cdot 10^{-5}$	6.15	1.02
	Singapore1_14	$2.53 \cdot 10^{-7}$	5.75	1.10		Japan27	$1.14 \cdot 10^{-7}$	5.81	0.96
	China1_D	$5.61 \cdot 10^{-7}$	6.67	0.81		USA1_737	$3.12 \cdot 10^{-7}$	4.72	0.81
	Germany7	$8.16 \cdot 10^{-7}$	6.80	0.72		Korea2_K	$6.53 \cdot 10^{-5}$	6.55	1.37
	Korea_4 7	$2.57 \cdot 10^{-6}$	6.85	0.44		USA2_106	$2.16 \cdot 10^{-6}$	6.22	0.79
	Korea_4 29	$1.40 \cdot 10^{-6}$	6.81	0.37		Singapore6	$7.16 \cdot 10^{-5}$	6.06	0.49
	USA2_407	$1.41 \cdot 10^{-7}$	6.19	1.00		USA2_1447	$1.15 \cdot 10^{-6}$	5.20	1.07
	USA2_4372	$1.13 \cdot 10^{-6}$	6.77	0.67		USA2_1257	$8.08 \cdot 10^{-6}$	6.39	0.41

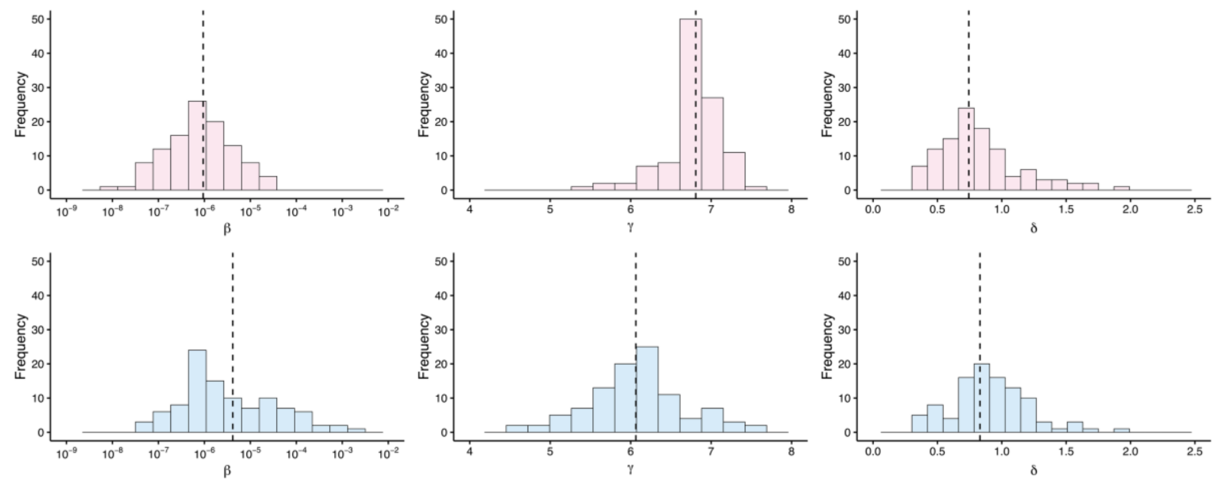

Figure S2. Distributions of the estimated parameters of the viral dynamics model. The top and bottom row shows the histograms of the estimated distribution of individual-level parameters (random effect) for symptomatic and asymptomatic patients, respectively. Vertical dashed lines represent the population-level (fixed effect) estimates for each parameter.

Conversion of viral load to infectiousness (and infectiousness threshold)

We defined "infectiousness" as the per contact probability of transmission. As observed in multiple epidemiological and experimental studies, the infectiousness becomes negligible when the viral load is below a threshold value (i.e., the infectiousness threshold)[7]. In this study, we assumed that an individual is infectious when the viral load is above the threshold, and non-infectious otherwise. The infectiousness was assumed

as logarithmically proportional to the viral load during the infectious period. We set the infectiousness threshold as $10^{5.0}$ copies/ml following epidemiological and experimental studies[8,9]. Specifically, the normalized infectiousness $u_i(t)$ for an individual i at time t after their infection was computed from viral load using the following equation:

$$u_i(t) = \begin{cases} A_i \cdot \log(V_i(t)), & t \in [t_{i,0}, t_{i,1}], \\ 0, & \text{otherwise}, \end{cases} \qquad (3)$$

where the viral load is above the infectiousness threshold from $t_{i,0}$ to $t_{i,1}$ (i.e., $V_i(t_{i,0}) = V_i(t_{i,1}) = 10^{5.0}$ copies/ml). The normalization constant A_i is defined as:

$$A_i = 1 \Big/ \sum_{t=t_{i,0}}^{t_{i,1}} \log V_i(t) \ . \qquad (4)$$

The length of the infectious period is defined as $t_{i,1} - t_{i,0}$, assumed to be contiguous because the viral dynamics is unimodal under reasonable parameter settings.

Simulation of incubation period using viral dynamics model

We assumed that the symptom starts during when the viral load is above $(100 - m)\%$ of the peak viral load. Specifically, the incubation period for an infected individual i was computed by the following process:

1) A longitudinal viral load data sample was generated by running the viral dynamics model with a parameter set randomly sampled from the estimated posterior parameter distributions. The peak viral load for an individual i is denoted by V_i^*.
2) We computed the time interval when the viral load is above $(100 - m)\%$ of the peak viral load, denoted by $[t_{i,0}^m, t_{i,1}^m]$. Note that $V_i(t_{i,0}^m) = V_i(t_{i,1}^m) = V_i^*(1 - m/100)$.
3) The incubation period for individual i (T_i) was then calculated as the sum of $t_{i,0}^m$ and a random number from an exponential distribution X: $X \sim Exp(\lambda)$: $T_i = t_{i,0}^m + X$.
4) Step 3) was repeated until the incubation period was in the interval $[t_{i,0}^m, t_{i,1}^m]$ and $[t_{i,0}, t_{i,1}]$: $T_i \in [t_{i,0}^m, t_{i,1}^m] \cap [t_{i,0}, t_{i,1}]$.

The two parameters, m and λ were determined so that the generated incubation period distribution matches the incubation period estimated by Hu et al. in an epidemiological study[10]: a Weibull distribution with mean 6.4 days (median: 5.7 and IQR: 3.2-8.8). Specifically, m and λ were computed to minimize the following sum squared error (SSE):

$$SSE(m, \lambda) = (Mean - 6.4)^2 + (Median - 5.7)^2 + (Q25\% - 3.2)^2 + (Q75\% - 8.8)^2,$$

where $Mean, Median, Q25\%\ and\ Q75\%$ are respectively the mean, median, 25% quantile and 75% quantile of generated distribution. Thus, we obtained an incubation period distribution with median 5.5 days and 50% interquantile range of 4.0-7.5 days.

Synthetic population

To model the contacts relevant for the transmission of SARS-CoV-2, we used the high-resolution multiplex network synthesized from real-world sociodemographic data that was developed in Mistry et al.[11]. This network consists of four layers representing contact settings: household, school, workplace, and community. The network features 200,000 households with about 500,000 individuals, corresponding to a representative subsample of the population of Indiana, USA. Each individual is assigned to a household, represented as a cluster in the household layer, and to the community, which assembles all individuals into a single cluster. Moreover, based on age-specific employment and school attendance probability, each individual can be assigned to a workplace (a cluster in the workplace layer) and a school (a cluster in the school layer). The community layer contains all individuals in a single cluster. Within each layer, clusters are connected such that all individuals within each cluster are connected, and no connections between individuals of different clusters exist.

VIBES model for SARS-CoV-2 transmission

In our individual-based model, SARS-CoV-2 spreads through contacts in the described network. At each time step during the simulation, an epidemiological status is associated to each individual: susceptible, latent, infectious pre-symptomatic, infectious symptomatic, infectious asymptomatic, and removed. Susceptible individuals can acquire the infection from infectious individuals that share a cluster with them. Once infected, a susceptible individual is assigned to the latent status, meaning that they carry the pathogen but cannot infect other individuals. Based on the estimated age-dependent probability of developing symptoms estimated by Poletti et al.[12] (see Table S2), we determine whether an infected individual will develop symptoms during the course of infection. If symptoms are to develop, we sample the viral dynamic parameters from the posterior distribution that was fitted to symptomatic patient data. Otherwise, we sample parameters from the distribution fitted to asymptomatic patient data. We then obtain a viral load trajectory by simulating the within-hosts model with the selected parameters.

Over the course of an infection, the viral load trajectory determines the progression of an individual through epidemiologic statuses. Specifically, when the viral load exceeds a threshold of 10^5 copies/ml[7], the individual becomes able to transmit the pathogen. At this point, individuals flagged to develop symptoms are assigned to the infectious pre-symptomatic status, while individuals who will not develop symptoms are assigned to the infectious asymptomatic status. When the time of symptom onset is reached, determined by the incubation period, infectious pre-symptomatic individuals are assigned to the symptomatic status. Finally, symptomatic and asymptomatic individuals are assigned to the removed status once their viral load goes below the same threshold of 10^5 copies/ml, at which point they are unable to infect other individuals and remain so until the end of the simulation.

The within-host viral dynamics also determine the infectiousness of an individual during their infectious period. The probability $p_{i \to j}(t)$ that infectious individual i infects

susceptible individual j that is in contact with i in setting $l \in \{\text{houehold, workplace, school, community}\}$ at time t is defined as:

$$\boldsymbol{p_{i \to j}(t) = \alpha \cdot \frac{\beta_l}{N_{i,l}} \cdot \; g_i \cdot \psi_i \cdot u_i(t - \tau_i) \cdot \Delta t,} \tag{5}$$

Where:

- α is the overall transmission rate;
- β_l is the layer-specific relative transmission risk;
- $N_{i,l}$ is the number of individuals in the given cluster of layer l;
- $u_i(t - \tau_i)$ is the infectiousness of individual i at step $t - \tau_i$, where τ_i represents the time of infection of individual i;
- g_i is a gamma-distributed random variable determining the overall infectiousness of individual i, with shape 0.235 and scale 4.26 to obtain the distribution of the number of secondary infections reported in Sun et al[13].
- ψ_i is the infectiousness of symptomatic individuals relative to asymptomatic individuals; namely, $\psi_i = 1$ if i is asymptomatic and $\psi_i = \psi$ if i is symptomatic.
- $\Delta t = 0.25$ days is the duration of a single time step in the simulation.

The rate α and the layer-specific transmission risks β_l, were set such that the reproduction number in the exponential phase was $R = 1.3$ and the proportions of infections in each layer matched those estimated by social settings, as reported in Liu et al.[14] (see Table S2). Each simulation was initialized with 30 latent individuals, with all other individuals susceptible. All model parameters are reported in Table S2.

Table S2. Model parameters. List of model parameters and values used in the main analysis with no interventions.

Parameter description	Parameter value
Study population	Indiana, USA
Number of individuals (population size)	502,697
Number of households	200,000
Length of a time step	0.25 days
Initial number of latent individuals	30
Setting-specific transmission risks (fraction of transmission by setting)[#]:<br>Households<br>Schools<br>Workplaces<br>Community	 1.0 {35%}<br>0.418 {15%}<br>0.307 {15%}<br>0.304 {35%}
Individual transmissibility coefficient[@]:<br>Gamma distribution's shape<br>Gamma distribution's scale	 0.24<br>4.13
Infectiousness of symptomatic individuals relative to asymptomatic individuals (ψ):	1
Probability of developing symptoms by age group[$]:<br>0-19 years old<br>20-39 years old	 18.1%<br>22.4%

40-59 years old	30.5%
60-79 years old	35.5%
80+ years old	54.6%

Transmission risks and fraction of transmission by setting refer to the scenario R=1.3, as estimated in Hu et al. [10] and Liu et al.[14]

@ Parameters set to obtain the distribution of the number of secondary infections reported in Sun et al.[13]

$ Reference: Poletti et al.[12]

Time window for the estimation of the reproduction number

From the synthetic line list of infections generated with VIBES, we can directly calculate the reproduction number of a simulation over time and for the entire simulation. The reproduction number is defined as the average number of secondary infections that a typical infected individual generates over the course of their infection, characterizing the overall infectiousness of the pathogen during one simulation. However, since the reproduction number decreases over time as immunity builds, we calculate the reproduction number only during early stages of each epidemic. The result allows us to compare different simulations in terms of the infectiousness.

We use thresholds in the cumulative incidence of infections to determine the window of calculation of the reproduction number. Given one simulation, the start of the calculation window w_0 is defined as the first time-step t in which the cumulative incidence of infections is greater than or equals to $c_0 = 1000$ individuals. The end of the calculation window w_1 is defined as the first time-step t in which the cumulative incidence of infections is greater than or equals to $c_1 = 7000$ individuals. The threshold c_1 for the end of the calculation window also defines the occurrence of an outbreak: If the infection incidence never reaches c_1 by the end of the simulation, we consider that it did not produce an outbreak, and the reproduction number is not calculated for that simulation. For this work, we only considered simulations that produce an outbreak for calculating the transmission pattern metrics.

All the presented results are based on a high-resolution grid of values of pathogen transmissibility. Specifically, we predefined a grid of values for the overall transmissibility (α) to simulate different values of the reproduction number. We then simulated 50 independent stochastic executions of the model for each α in the pre-selected grid. For each value of α, we calculated the mean values of R, T_g, S_I and P_T from the ensemble of independent executions. Finally, to estimate the uncertainty on each epidemiological metric, we used a quadratic spline interpolation with R as the independent variable and the desired parameter (T_g, S_I or P_T) as dependent variable, such that R lies within the target range. Confidence intervals represent quantiles of this interpolated ensemble.

For the estimation of the epidemiological metrics derived from the literature (i.e., those used to validate model estimates), we used an unweighted average of the estimates reported in each study. The uncertainty provided on these estimates represents the interquartile range of the means.

Supplementary Results

Estimation of uncertainty

Since VIBES keeps track of the entire transmission chains; we can use this information to directly calculate the epidemiological metrics of interest (e.g., reproduction number, generation time) both at the individual and population level.

Figure 2 in the main text shows that population-level estimates of the generation time, serial interval, and fraction of presymptomatic transmission have relatively narrow confidence intervals. However, individual-level estimates show a remarkable variability (Fig.S3). For example, considering a random sample of 12 infected agents that generated at least 1 secondary infection, we found a generation time ranging from 3.22 to 13.25 days, serial interval from 0.25 to 8.88 days (note that negative values are allowed, but they did not appear in our random sample), and fraction of pre-symptomatic infections ranged from 0 to 100%, as it is typically calculated on a small number of secondary cases, making the 0 and 100% as the two most likely outcomes.

The variability at individual level causes population-level epidemiological metric estimates to depend on the sample size used for estimation. In the main text, we showed model estimates of metrics using the entire transmission chains (which includes tens to hundreds of thousands of transmission events depending on the simulated scenario), which resulted in a relatively narrow confidence interval. To emulate the conditions of a typical empirical field studies, we estimated the same key epidemiological metrics using subsamples of infectors from VIBES simulations. Specifically, we randomly selected N infection pairs from the simulation, where N is a specified sample size, then calculated the mean generation time, serial interval, and fraction of pre-symptomatic transmission from the subsample. This sampling procedure was repeated $M = 1000$ times, creating a bootstrap confidence interval. We used $N \in \{10, 25, 50, 100, 250, 500\}$, consistent with the sample sizes reported in the field epidemiological studies included in our analysis. For small sample sizes (e.g. $N < 50$), our estimates have bootstrap confidence intervals similar to the variability that we found when combining field epidemiological studies (Fig. S4).

Finally, it is important to stress that estimates reported in field epidemiological studies are highly affected by the deployed public health interventions. During the early phase of the pandemic (i.e., the period of interest for the included studies), a vast array of public health interventions was deployed, and it kept evolving over time, ranging from case isolation to school and workplace closures, masking, partial lockdowns, etc. Figure S4 shows VIBES estimates of generation time, serial interval, and fraction of pre-symptomatic transmission when we combine results from simulations without interventions and from simulations with household isolation of symptomatic individuals. Our results show much wider confidence intervals, in line with the variability in field epidemiological studies.

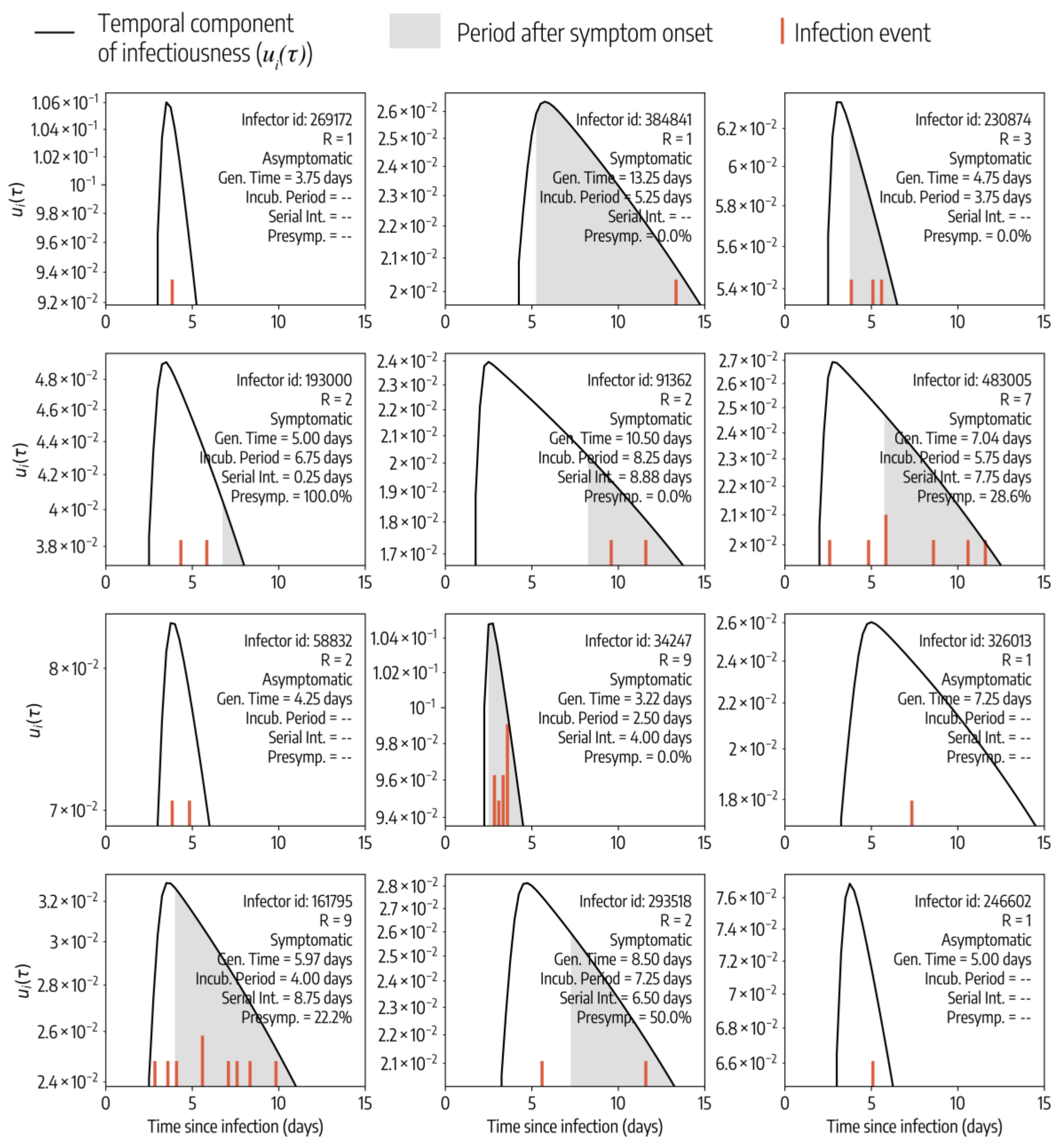

Figure S3. Individual-level metrics for randomly selected infected agents. Each panel represents a single infectious agent in VIBES, which was randomly selected among the pool of infected individuals that generated at least 1 secondary infection taken from a simulation with an overall reproduction number $R = 1.3$. The solid black line represents the temporal component of the infectiousness of individual i ($u_i(\tau)$). Red bars represent secondary infection events, where the height of the bar represents the number of individuals that i infected at that time step of the simulation. For symptomatic individuals, the gray shaded area represents the period after symptom onset. The text within each panel reports individual-level epidemiologic metrics for that individual i: number of secondary infections (R), whether i was symptomatic or asymptomatic, generation time, incubation period, serial interval, and fraction of pre-symptomatic transmission.

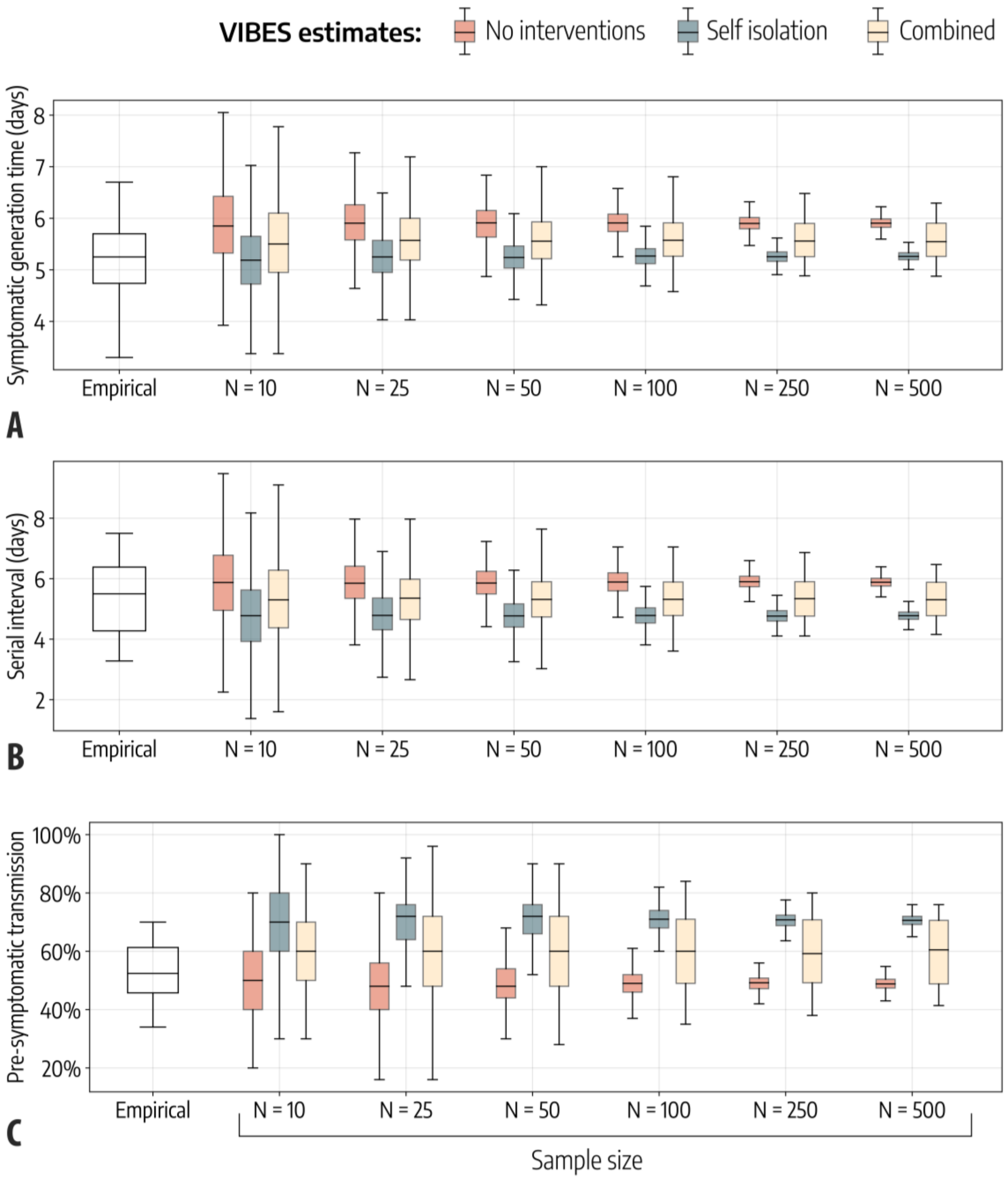

Figure S4. Estimated epidemiological metrics and sample size. **A** Boxplots of the distribution of the generation time for symptomatic individuals by combining the identified field epidemiological studies (same as in Fig. 2 in the main text) and as estimated by VIBES using a subsample of infections from a scenario with $R = 1.3$ with no interventions, home isolation of symptomatic individuals, and combining both no interventions and home isolation. For model simulations, the box represents the 50% interquantile range of the bootstrapped estimates, the horizontal line represents the median, and the whiskers represent the farthest data point within 1.5 times the inter-quartile range from the box. **B** Same as A for the serial interval. **C** Same as A for the fraction of pre-symptomatic transmission.

Epidemic trajectories

Simulations using the VIBES model typically show a single-peak outbreak pattern infection incidence over time. With a fixed set of parameters, we performed $n = 50$ simulations and calculated the number of new infections at each time step. Since the length of the time step is $\Delta t = 0.25$ days, we aggregated the number of infections at each day to obtain a daily time series of the infection incidence. We then shifted the trajectory of each simulation such that time $t = 0$ represents the first time step in which the cumulative infection incidence was greater than 1 infection per 1000 individuals. With all simulations aligned in such way, we calculated the daily average incidence, as well as the daily 95% interquantile range of the incidence. We obtained simulations for different values of the reproduction number by changing the scaling rate α. The results indicate that simulations display a typical single-peak outbreak pattern, which reproduces the patterns obtained with traditional between-hosts simulations when immunity wane is not considered (Fig. S5). This shows that our model is able to reproduce patterns of other models that have been successfully employed to simulate infectious disease outbreaks.

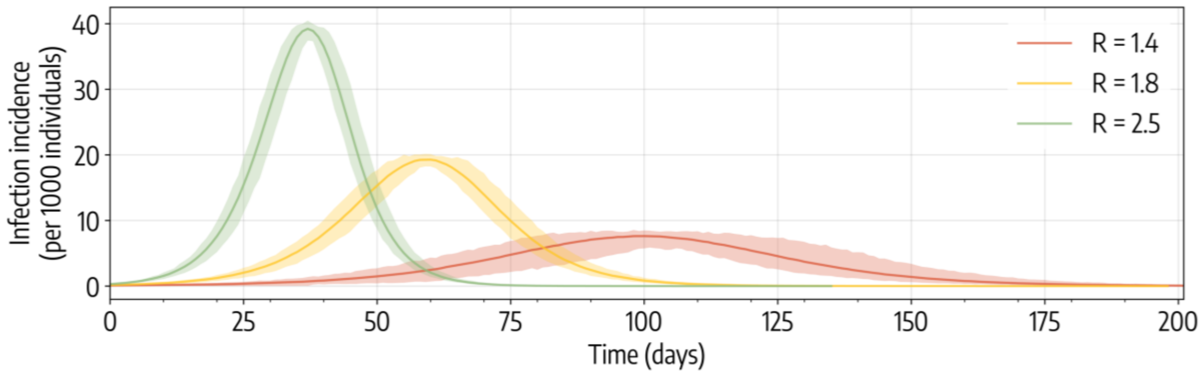

Figure S5. Infection incidence over time for different values of the reproduction number. Average number of new infection over time during simulations with three different reproduction number values. The trajectory of each simulation is first aligned by setting $t = 0$ as the first day in which the cumulative incidence is greater than 1 infection per 1000 individuals. The shaded areas represent the 95% interquantile ranges of the infection incidence.

Sensitivity analysis: Probability of symptom development

We tested different assumptions for the probability that each infected individual develops symptoms. This was achieved by multiplying the age-dependent probabilities described in Table S2 by a uniform factor of 1.5 (50% higher probability of symptom development) and 0.5 (50% lower probability of symptom development). The estimates for the generation time of symptomatic and asymptomatic infectors were robust to changes in this assumption. The overall generation time shifts towards lower values when the symptom development probability is higher. This happens because symptomatic individuals, on average, have lower generation time than asymptomatic ones, and therefore increasing the ratio of symptomatic infections over asymptomatic ones biases the generation time towards lower values. Similarly, the overall generation time shifts towards higher values when the probability of symptom development is lower. No significant effects of changing the symptom development probability were observed into the serial interval and the pre-symptomatic transmission (Fig. S6).

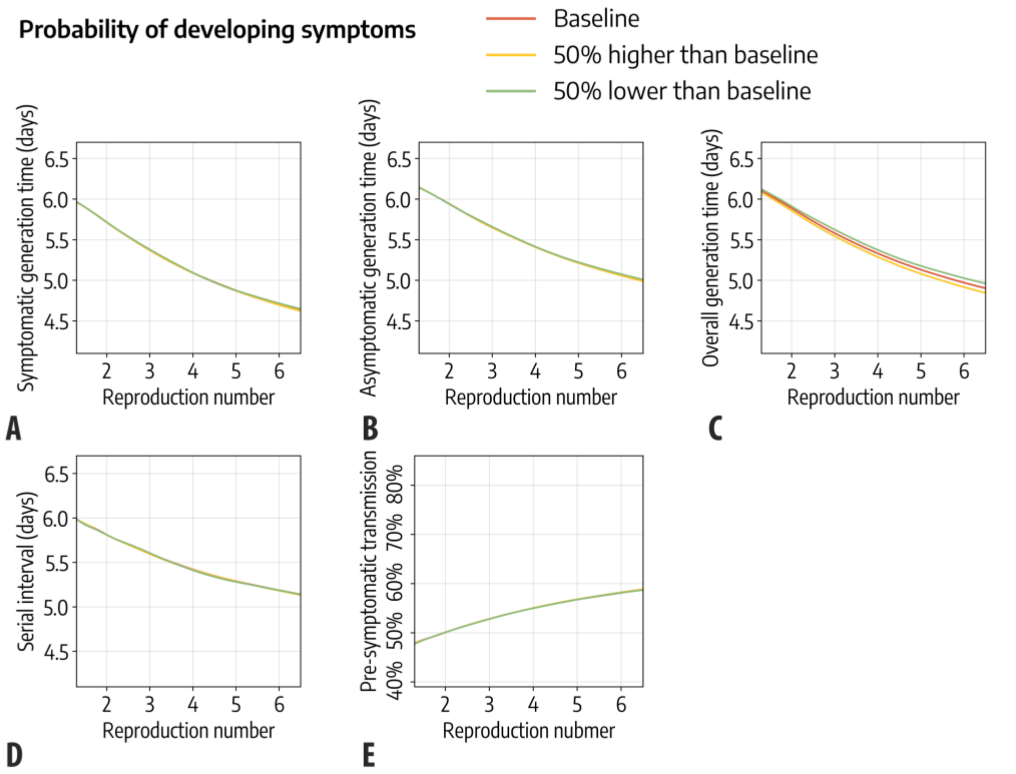

Figure S6. Probability of symptom development and its effect on transmission metrics. Average generation time for symptomatic individuals estimated by VIBES, assuming the baseline probability of symptom development[12], as well as a 50% higher probability (yellow curve) and a 50% lower probability (green curve). **B** As A but for the generation time for asymptomatic individuals. **C** As A but for the generation time for all infected individuals. **D** As A but for the average serial interval. **E** As A but for the proportion of pre-symptomatic transmission.

Sensitivity analysis: Infectiousness of symptomatic individuals relative to asymptomatic ones

Our results are also robust to variations in the infectiousness of symptomatic individuals when compared to asymptomatic ones. To study that, we increased the infectiousness of pre-symptomatic and symptomatic individuals (ψ_i) to 3 and 5 times higher than that of asymptomatic individuals. This led to only marginal changes in the generation time, serial interval and fraction of pre-symptomatic transmission, showing the transmission patterns that we studied are not affected by this assumption. We also investigated the ratio of secondary cases generated by symptomatic infectors relative to asymptomatic ones, which increases as the infectiousness of symptomatic individuals relative to asymptomatic ones increases (Fig. S7).

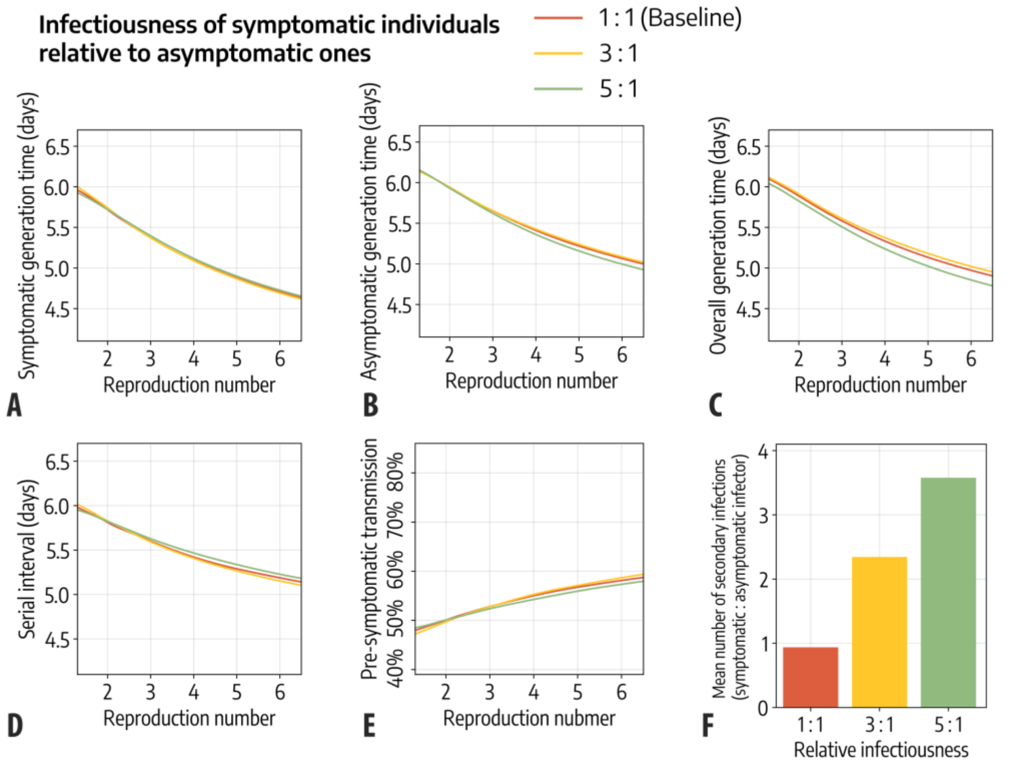

Figure S7. Effect of infectiousness of symptomatic individuals relative to asymptomatic ones and its effect on transmission metrics. **A** Average generation time for symptomatic individuals estimated by VIBES, assuming the baseline infectiousness of symptomatic and asymptomatic individuals, as well as assuming that symptomatic individuals transmit 3 times (3:1 ratio) and 5 times (5:1 ratio) more than asymptomatic individuals. **B** As A but for the generation time for asymptomatic individuals. **C** As A but for the generation time for all infected individuals. **D** As A but for the average serial interval. **E** As A but for the proportion of pre-symptomatic transmission. **F** Mean number of secondary infections generated by symptomatic infectors relative to asymptomatic ones as a function of the relative infectiousness (i.e., infectiousness of symptomatic individuals relative to asymptomatic ones).

Sensitivity analysis: Distribution of the numbers of secondary infections

Transmission of SARS-CoV-2 shows significant overdispersion[13], meaning that the distribution of the number of secondary infections has a negative-binomial shape, with superspreading events likely. In the baseline model, we applied a gamma-shaped distributed coefficient to the infectiousness of each individual which, convolved with the transmission process, creates a negative binomial distribution similar to the one observed in the field. As a sensitivity analysis, we removed this individual coefficient, which results in a Poisson-shaped distribution of secondary cases. This caused small changes in the estimated epidemiologic metrics, while the overall trend remained the same. The Poisson-distributed infectiousness implied shorter generation times for both symptomatic and asymptomatic individuals, as well as shorter serial intervals and larger pre-symptomatic transmission (Fig. S8).

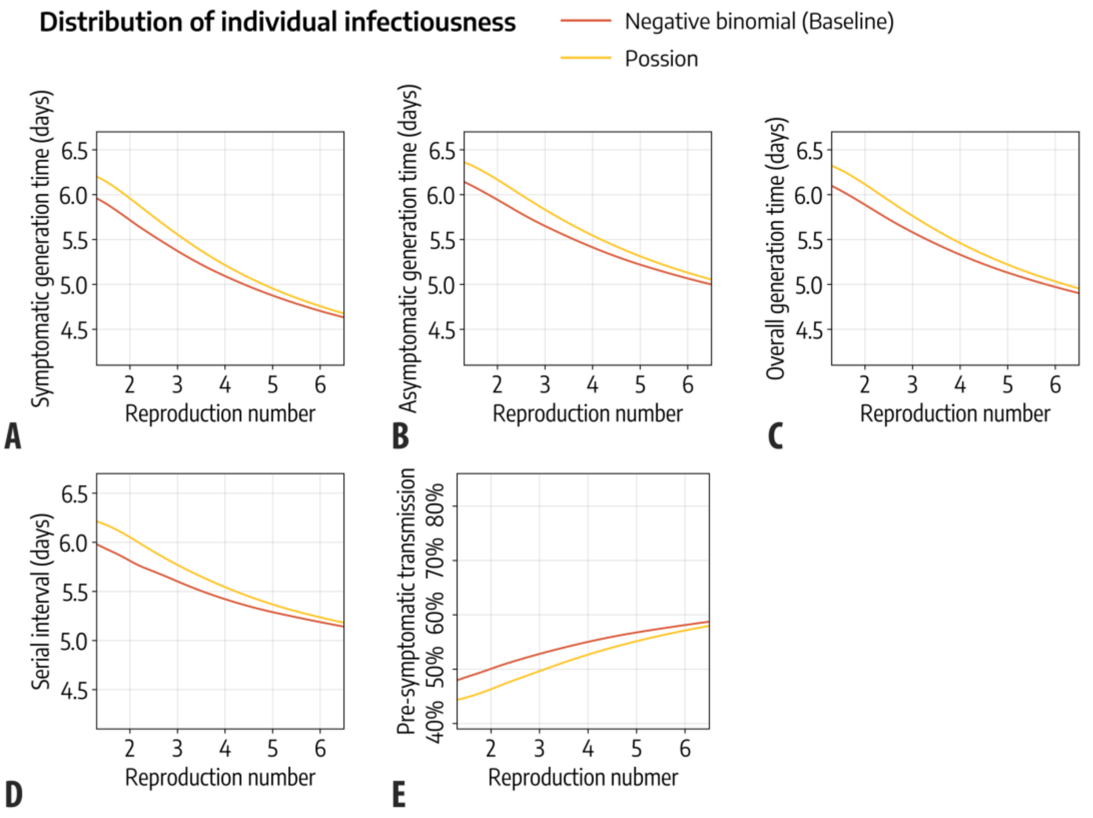

Figure S8. Distribution of the number of secondary cases and its effect on transmission metrics. A Average generation time for symptomatic individuals estimated by VIBES, having the baseline distribution of number of secondary cases (negative binomial with k ~= 0.5), as well as assuming that there is no overdispersion for the distribution of secondary cases (Poisson). **B** As A but for the generation time for asymptomatic individuals. **C** As A but for the generation time for all infected individuals. **D** As A but for the average serial interval. **E** As A but for the proportion of pre-symptomatic transmission.

References

1. Jeong, Y. D. *et al.* Designing isolation guidelines for COVID-19 patients with rapid antigen tests. *Nat. Commun.* **13**, 4910 (2022).

2. Ikeda, H. *et al.* Quantifying the effect of Vpu on the promotion of HIV-1 replication in the humanized mouse model. *Retrovirology* **13**, 23 (2016).

3. Kim, K. S. *et al.* A quantitative model used to compare within-host SARS-CoV-2, MERS-CoV, and SARS-CoV dynamics provides insights into the pathogenesis and treatment of SARS-CoV-2. *PLOS Biol.* **19**, e3001128 (2021).

4. Goyal, A., Cardozo-Ojeda, E. F. & Schiffer, J. T. Potency and timing of antiviral therapy as determinants of duration of SARS-CoV-2 shedding and intensity of inflammatory response. *Sci. Adv.* **6**, eabc7112 (2020).

5. Jeong, Y. D. *et al.* Revisiting the guidelines for ending isolation for COVID-19 patients. *eLife* **10**, e69340 (2021).

6. Monolix Suite. *Lixoft* https://lixoft.com/products/monolix/.

7. Zou, L. *et al.* SARS-CoV-2 Viral Load in Upper Respiratory Specimens of Infected Patients. *N. Engl. J. Med.* **382**, 1177–1179 (2020).

8. Wölfel, R. *et al.* Virological assessment of hospitalized patients with COVID-2019. *Nature* **581**, 465–469 (2020).

9. van Kampen, J. J. A. *et al.* Duration and key determinants of infectious virus shedding in hospitalized patients with coronavirus disease-2019 (COVID-19). *Nat. Commun.* **12**, 267 (2021).

10. Hu, S. *et al.* Infectivity, susceptibility, and risk factors associated with SARS-CoV-2 transmission under intensive contact tracing in Hunan, China. *Nat. Commun.* **12**, 1533 (2021).

11. Mistry, D. *et al.* Inferring high-resolution human mixing patterns for disease modeling. *Nat. Commun.* **12**, 323 (2021).

12. Poletti, P. *et al.* Association of Age With Likelihood of Developing Symptoms and Critical Disease Among Close Contacts Exposed to Patients With Confirmed SARS-CoV-2 Infection in Italy. *JAMA Netw. Open* **4**, e211085 (2021).

13. Sun, K. *et al.* Transmission heterogeneities, kinetics, and controllability of SARS-CoV-2. *Science* **371**, eabe2424 (2021).

14. Liu, Q.-H. *et al.* Model-based evaluation of alternative reactive class closure strategies against COVID-19. *Nat. Commun.* **13**, 322 (2022).
